# Multi-Sensor Fusion and Error Compensation of Attitude Measurement System for Shaft Boring Machine

**DOI:** 10.3390/s19225007

**Published:** 2019-11-16

**Authors:** Xinliang Wang, Jiangong Liu, Yang Liu, Wenjun Fu, Lei Zhu

**Affiliations:** 1 School of Mechanical Electronic and Information Engineering, China University of Mining and Technology, Beijing 100083, China; liuyangebox@126.com; 2 College of Mechanical and Equipment Engineering, Hebei University of Engineering, Handan 056038, China; liujg@jzeg.cn; 3 Tiandi Science and Technology Co., Ltd., Beijing 100013, China; Fuwj6688@163.com; 4 CCTEG Shenyang Research Institute, Fushun 113122, China; zhulei-nuli@163.com

**Keywords:** shaft boring machine (SBM), attitude measurement, multi-sensor fusion, dual coordinate method, complementary filter, extended Kalman filter (EKF)

## Abstract

To ensure that the shaft boring machine (SBM) runs along the pre-designed axis steadily, the role of the attitude measurement system is essential, but its accuracy and reliability cannot be guaranteed. Currently, there is no effective technology to meet the actual requirements, and there is a lack of relevant theoretical research in this field. Through further study of the attitude analysis method and multi-sensor fusion technology, this paper presents a dual coordinate method, which can be used to describe the attitude characteristics of the SBM. Moreover, this paper discusses the relationships between the attitude changes and the values of the angle as well as displacement and analyzes the implementation complexity and computational efficiency of related algorithms in software and hardware. According to the working characteristics of the SBM, the hardware design and the reasonable layout of the attitude measurement system are provided. Based on multi-sensor data, this paper puts forward an improved method combining a complementary filter with an extended Kalman filter (EKF) for attitude estimation and error compensation. The simulation experiments of different working processes verify the steady-state response and dynamic response performance of the method. Experimental results show that the dual coordinate method and the proposed filter are more suitable for attitude estimation of the SBM compared to other methods.

## 1. Introduction

**Figure 1:**
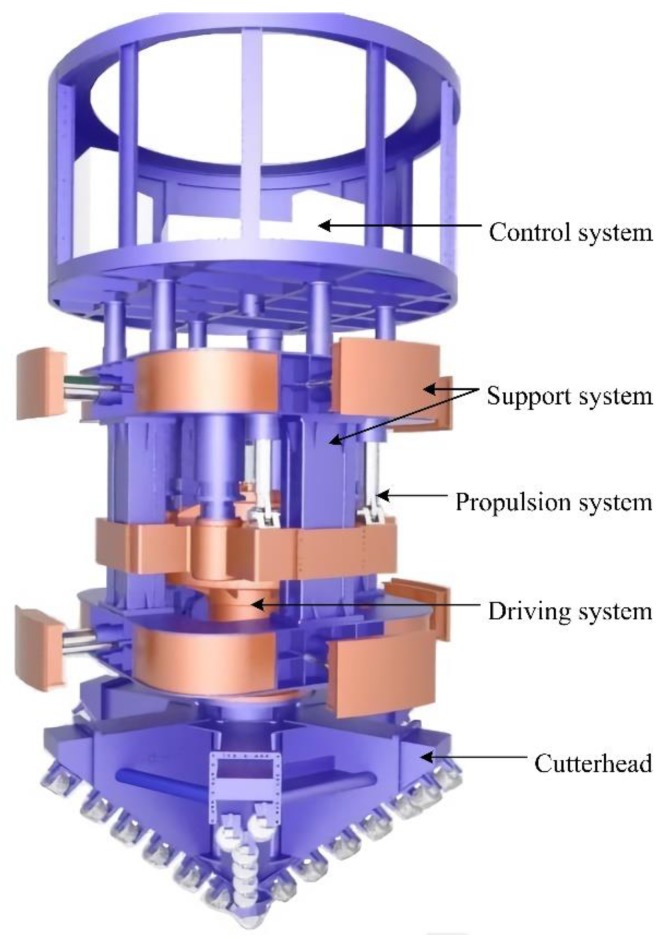
Overall structure of the shaft boring machine (SBM).

A shaft boring machine (SBM), as a special type of shaft drilling equipment, can carry out mechanical rock breaking and support quickly. It has many advantages, such as a high degree of mechanization without blasting operation, fewer underground workers, high construction efficiency, good completion quality, and high security. In fact, it has become the major piece of technical equipment for infrastructure construction and resource development, as well as an important development direction of intelligence and automation for modern mines [1,2]. The SBM is composed of a control system, support system, propulsion system, driving system, and cutterhead [3,4]; the overall structure is shown in Figure 1. The control system is the core part, and it is responsible for coordinating the normal work of other parts and completing the data interaction with a remote monitoring center. Because the SBM adopts the rolling impact and shear mode to break the complex rock structure, the vibration is strong, and it has a small operating space, high temperature, high dust concentration, and serious electromagnetic interference, and these factors have a serious influence on the control system [5,6]. In particular, the movement track of the SBM is easy to deviate from the shaft design axis, and the trajectory is out of alignment, which has a direct impact on construction efficiency and quality and even causes safety accidents. Therefore, attitude measurement units are the key part of the control system, and they are extremely important for the SBM working safely and stably.

At present, attitude measurement methods of tunnel boring machines applied in underground construction contain manual methods and automatic methods [7]. The operation time is long, and the calculation is complicated for manual methods, but the manual methods are often used as supplementary means for an automatic measurement method because of their high detection accuracy. Automatic measurement methods mainly include a gyroscope guidance system, prism guidance system, laser target guidance system, and visual measurement guidance system, and these systems have some advantages and disadvantages. TMG-32B (gyroscope guidance system) is low cost but needs regular calibration due to the poor stability and low measurement accuracy. ROBOTEC, PPS, and RMS-D (prism guidance system) have a simple structure and low cost but have high requirements for the installation environment with poor real-time performance and low precision. ZED and TUnIS (laser target guidance system) have long measurement distance and high precision but easily cause errors and improper installation. The visual measurement guidance system has a large field of view and good real-time performance, but it is still in the research stage and has some limitations, so there is no typical engineering application [8,9,10,11,12].

Obviously, different attitude measurement methods have their applicability and limitations, and the working environments of the SBM are complicated, including mechanical vibration, electromagnetic interference, temperature effect, pressure change, and so on. Consequently, there are no effective technical means for the attitude measurement of the SBM, and it is easy to generate cumulative and random errors only by a single attitude measurement method [13,14]. To avoid the above problems, multi-sensor fusion technology can be used to study attitude calculation and error compensation methods, and thus can improve the accuracy and reliability of the attitude measurement system.

Multi-sensor fusion technology is first applied in strategic early warning, precision guidance, and other military fields, and it is gradually extended to remote sensing, medical diagnosis, wireless communication, industrial control, fault diagnosis, and other civilian fields. Currently, the common data fusion algorithms mainly include the weighted average method, Bayesian estimation method, complementary filter method, fuzzy logic inference method, wavelet transform method, artificial neural network method, gradient descent method, and Kalman filter method [15,16]. Multi-sensor fusion technology can avoid lots of problems existing in the single sensor system, enhance the viability of related systems, expand time and space coverage, improve credibility, reduce the ambiguity of information, and enhance the robustness and reliability of total system [17]. At the same time, the accuracy, applicability, and fast response of the multi-sensor data fusion method are important reference indexes, especially the implementation complexity and computational efficiency of different algorithms in software and hardware. In these aspects, the complementary filter and Kalman filter have certain advantages compared with other methods [18,19,20], but the related theories and implementation processes still need further study.

The sensors of micro electro mechanical system (MEMS) have advantages of small size, low power consumption, low weight, low cost, and so on, and they have been widely used in fields of spacecraft, unmanned aerial vehicles (UAVs), mobile robots, pedestrian navigation, and vehicle attitude measurement and control [21,22]. Nevertheless, due to their manufacturing processes and working principles, inertial measurement units (IMU) based on MEMS have large systematic errors, such as deviation, scale factor, and drift. Moreover, the installation mode, temperature, vibration, impact, and noise also cause random errors [23,24], so the accurate attitude measurement can be realized only after signal processing. Nekrasov et al. [25] and Lall et al. [26] analyzed the failure mode of the MEMS gyroscope by thermal cycle and vibration tests and improved the performance of the gyroscope with modification tests. However, the method tends to have poor versatility only through hardware and mechanical structure improvements because there are differences between different MEMS sensors. Therefore, it is necessary to study the data fusion and error compensation method based on MEMS multi-sensors to effectively improve their applicability in the attitude measurement of the SBM.

In the field of underground space exploration, there are few studies on the data fusion and attitude estimation of MEMS sensors. Because of the complexity and particularity of the SBM, there is no reliable solution, and yet, some research results in other fields are worthy of reference. In the application of aircraft navigation, the noise and drift of MEMS inertial sensors will produce large errors in the attitude and heading reference system (AHRS), which can be improved by some sensor fusion methods, such as the Kalman filter [27]. The adaptive Kalman filter [28] and extended Kalman filter (EKF) [29] with a linear model and adaptive gain were adopted to avoid non-linear problems of conventional methods and the influence of dynamic acceleration on filter performance. Furthermore, the information fusion and attitude estimation based on the non-linear observer of a quaternion is a further improvement for the Kalman filter [30]. Zhu et al. [31] carried out real-time measurement and state estimation for the attitude and stability of articulated heavy vehicles by MEMS sensors and the complementary filter, which improved the safety and stability of the vehicle. Actually, the complementary filter method has advantages of having simple principles and small computation, but its disadvantage is that there is no reliable method to identify the boundary between high-pass filtering and low-pass filtering. The Kalman filter has a limited ability to adjust the changes of acceleration and vibration, but MEMS sensor fusion can restrain abnormal changes of external parameters. Generally, the accuracy is not ideal only through the simple integration of a gyroscope, accelerometer, and magnetometer [32], but it is feasible to compensate the drift errors of the gyroscope with data from the accelerometer and magnetometer.

At the same time, to reduce the data acquisition and calculation burden of remote control centers and improve the ability of autonomous attitude measurements and rapid response for the SBM, it is necessary to implement the algorithm in attitude measurement units and the embedded processor of the monitoring terminal, but the implementation complexity and computational efficiency of the hardware and software must be considered. Due to the speed limit, the 8-bit and 16-bit microcontrollers only support fixed-point operation, have some difficulties in algorithm implementation, and can barely run some simple algorithms [33,34]. However, the flexibility of the algorithm is obviously improved on advanced RISC (reduced instruction set computing) machine (ARM), field programmable gate array (FPGA) and other high-speed chips. For instance, the STM32F3 evaluation board can process the gradient descent method, nonlinear complementary filter, and standard EKF method quickly [35]; the STM32F103 controller can implement EKF based on quaternion, which meets the accuracy requirement for the attitude measurement of UAVs in dynamic environments [36]; Sabatelli et al. [37] proposed an IMU sensor fusion method based on a double stage Kalman filter to reduce the complexity of the algorithm, which was verified on FPGA.

**Figure 2:**
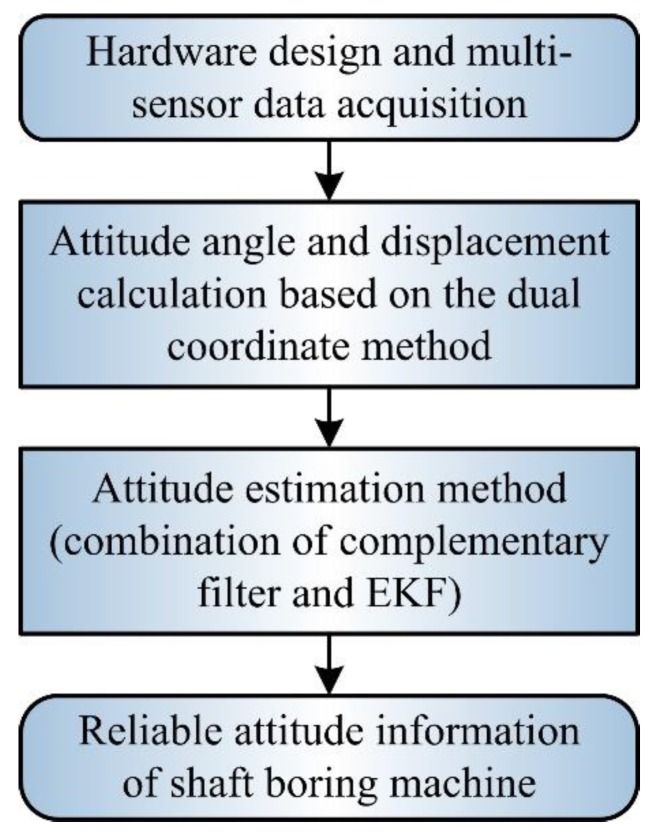
Block diagram of multi-sensor fusion and error compensation.

To solve the above problems, based on hardware design and multi-sensor data acquisition, this paper presents a dual coordinate method suitable for the SBM. The method reflects the working characteristics of the SBM and external environment and can be used to calculate the attitude angle and displacement. Combining the complementary filter with EKF, a fusion method of multi-sensor data is put forward to improve the accuracy of attitude estimation, and the method can effectively compensate estimation errors under working conditions. Meanwhile, the theoretical analysis and simulation experiments are adopted to validate related methods. Finally, the reliable attitude information of the SBM is obtained. The block diagram of multi-sensor fusion and error compensation is shown in Figure 2.

Thus, the main contributions of the presented work are (i) a dual coordinate system structure and analysis method suitable for the attitude measurement of the SBM, (ii) a fusion method of attitude estimation and error compensation based on the complementary filter and EKF, (iii) the complexity and computational efficiency analysis of different algorithms to ensure the real-time performance and reliability of data fusion results.

The rest of this paper is structured as follows. Firstly, Section 2 introduces the hardware composition and design of the attitude measurement system for the SBM. Section 3 presents the dual coordinate method based on angle and displacement and then analyzes the attitude characteristics of the SBM, matrix transformation, and the relationship between the attitude changes and coordinate values. The implementation processes of the improved attitude estimation algorithm are explained in Section 4. Next, experimental results are presented and discussed in Section 5. Finally, the conclusions and future work are proposed in Section 6.

## 2. Hardware Design

**Figure 3:**
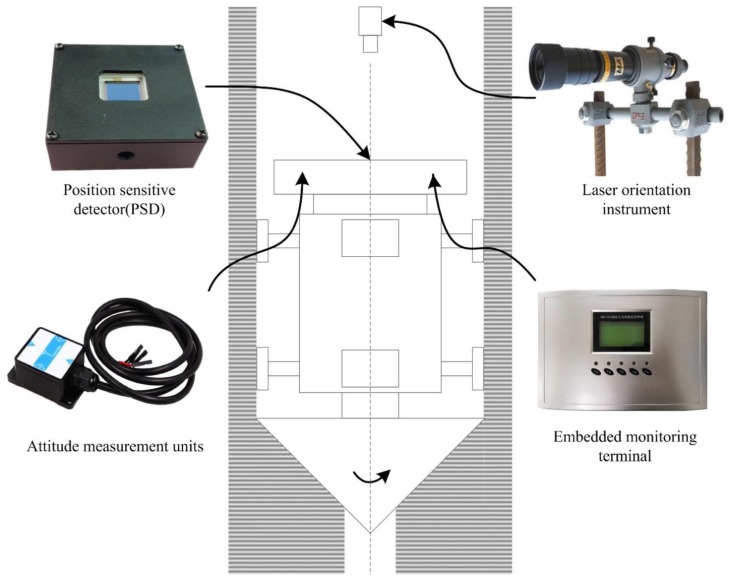
Hardware composition of the attitude measurement system.

**Figure 4:**
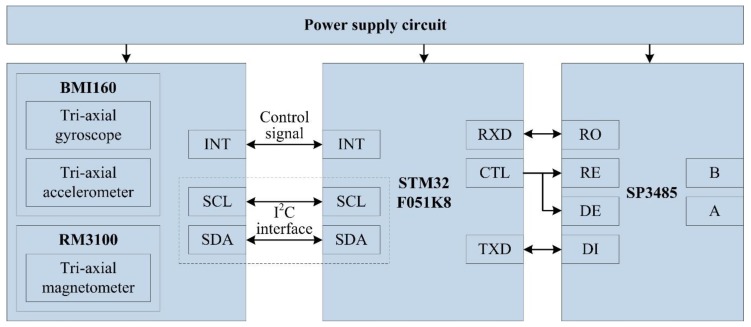
Composition of the attitude measurement unit.

**Figure 5:**
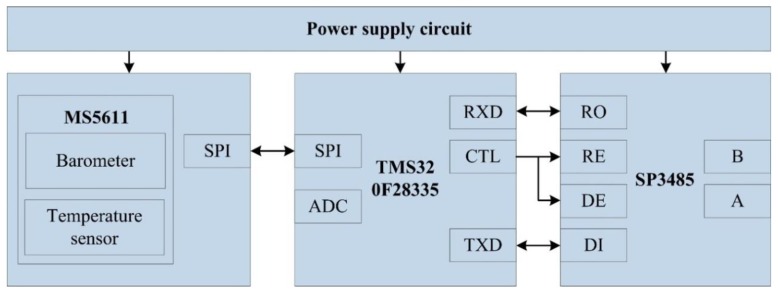
Composition of embedded monitoring terminal.

This section mainly introduces the hardware architecture of the attitude measurement system for the SBM. As shown in Figure 3, the SBM is moving down along the rock structure (shadow part). To ensure the running track of the SBM, the attitude measurement system generally includes a laser orientation instrument, position sensitive detector (PSD), attitude measurement units, and an embedded monitoring terminal. The technical characteristics of each component are as follows:

1. Laser orientation instrument (YHJ800, Beijing Wokesida Technology Development Co., Ltd., Beijing, China), which is fixed vertically on the sidewall of the shaft. At the first time, it locates 5 m above the SBM and is used to point the direction of the drilling construction. The low power semiconductor laser is driven by a DC power supply to generate the laser beam; the laser power is more than 10 mW, and the effective range of laser is 800 m.
2. Position sensitive detector (PSD6060, Shanghai Ouguang Electronic Technology Co., Ltd., Shanghai, China), which is installed on the top surface of the SBM, and it receives the laser signal to determine the horizontal relative displacement of the SBM. It is a two-dimension device, and the photosensitive surface range is 60 × 60 mm. Based on the photoelectric effect of the PIN photodiode, it can convert the light point position at the photosensitive surface to an electrical signal and has high position resolution and fast response speed. The output signal is only related to the light energy center position, independent of spot size and shape.
3. Attitude measurement units are fastened at the measuring point on the top of SBM, each unit integrates an embedded controller and MEMS sensors (a gyroscope, accelerometer, magnetometer, etc.), and the units are applied for preliminary data fusion to obtain the deviation angle of the SBM. As shown in Figure 4, each unit is composed of a power supply circuit, microcontroller, gyroscope, accelerometer, magnetometer, and 485 communication module SP3485 (MaxLinear, INC., Carlsbad, CA, USA). The STM32F051K8 (STMicroelectronics, Geneva, Switzerland) is the core part, and its operating frequency is up to 48 MHz. The signals of BMI160 (Bosch Sensortec GmbH, Reutlingen, Germany, including a tri-axial gyroscope and a tri-axial accelerometer) and RM3100 (PNI Sensor Corporation, Santa Rosa, CA, USA, a tri-axial magnetometer) are collected by interface I^2^C (SCL and SDA). The interrupt control signal is issued by the custom interface INT of STM32F051K8, and the data transfer interrupt is triggered by the interface INT of BMI160. Meanwhile, the chip pin CTL is also the custom interface of STM32F051K8, and it can control the enable signal of sending and receiving for interface RE and DE of SP3485. Moreover, RXD is the data receiving interface and receives data from the interface RO, TXD is the data sending interface and sends data to the interface DI, and then attitude measurement units communicate with the embedded monitoring terminal through the 485 bus (interface A and B).
4. The embedded monitoring terminal is on the top console of the SBM and collects information, such as displacement, air pressure, temperature, deviation angle, and then completes secondary data fusion and the attitude calculation. As shown in Figure 5, it includes a power supply circuit, microcontroller, air pressure sensor, 485 communication unit (SP3485), and so on. The core microcontroller is TMS320F28335 (Texas Instruments Incorporated, Dallas, TX, USA), and the operating frequency of the DSP (digital signal processor) controller is up to 150 MHz. TMS320F28335 receives the signal from MS5611 (Measurement Specialties, Inc., Hampton, VA, USA, including a barometer and a temperature sensor) with the interface SPI, and acquires the displacement value of the PSD by the interface ADC, the definition and function of 485 communication interface are similar to the attitude measurement unit. Meanwhile, the embedded monitoring terminal receives the data transmitted by attitude measurement units and carries out data interaction with attitude measurement units, the PLC (programmable logic controller), and the remote control center with the 485 bus (interface A and B).

## 3. Dual Coordinate Method

**Figure 6:**
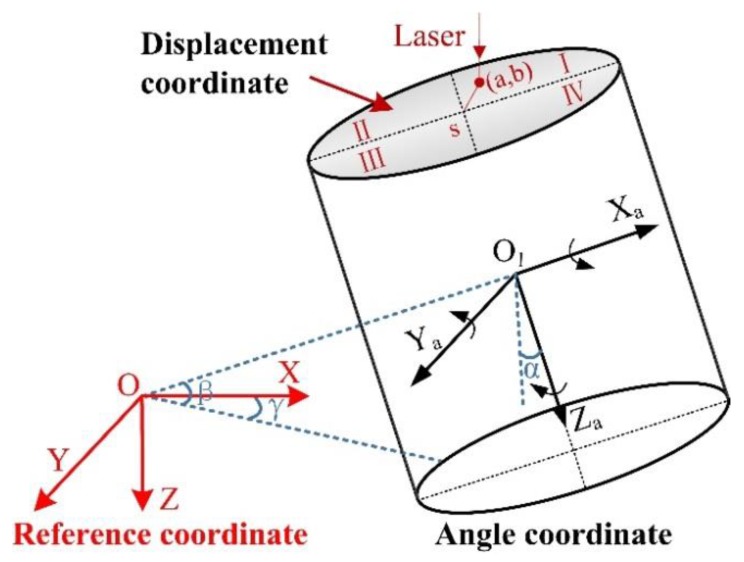
Dual coordinate system of the SBM (angle and displacement).

To describe the spatial motion state of the SBM, the reference coordinate system and target coordinate system must be established. Just like the navigation system of UAV, it has a coordinate navigation system (north-east-down) and body coordinate system, can calculate the angle deflection, and acquire the location information through GPS, COMPASS, and GLONASS [38], which are not suitable for the shaft environment. Therefore, according to the working characteristics of the SBM, on the one hand, the angle analytical coordinate system needs to be established to obtain the angle deflection; on the other hand, the displacement coordinate system must be established based on the PSD and laser orientation instrument to obtain the horizontal displacement of the SBM relative to the shaft design axis. As shown in Figure 6, combined with the calculation data of the dual coordinate system, the accurate attitude information of the SBM can be determined in the restricted space of the shaft.

### 3.1. Angle Coordinate Analysis

Attitude angles are three-dimensional vectors used to determine the rotation of an object on its axis [39]; they represent the rotation angle of the SBM around three axes of the coordinate system. According to the relationship between reference coordinate and angle coordinate, the attitude of the SBM relative to the horizontal plane is obtained. As shown in Figure 6, the attitude angles of the SBM include roll angle *α*, pitch angle *β*, and yaw angle *γ*. The specific definitions are as follows:

1. Roll angle *α*: the angle between the *Z_a_* axis and the plumb plane containing the *X_a_* axis after the SBM rotates around the *X_a_* axis. It is positive to the right and negative to the contrary, which shows the deflection degree around the *X_a_* axis; the threshold range is [−15°, 15°].
2. Pitch angle *β*: the angle between the *X_a_* axis and the horizontal plane after the SBM rotates around the *Y_a_* axis. When the positive half of the *X_a_* axis is above the horizontal plane, the pitch angle is positive, otherwise it is negative, which proves the deflection degree around the *Y_a_* axis; the threshold range is [−15°, 15°].
3. Yaw angle *γ*: the angle between the projection of the *X_a_* axis on the horizontal plane and the *X_a_* axis of the reference frame after the SBM rotates around the *Z_a_* axis. When the *X* axis rotates clockwise to the *X_a_* axis projection, the yaw angle is positive, otherwise it is negative. It is 0° under normal conditions, non-zero indicates that the support system has failed or the SBM is passing through an abnormal geological area, which causes the SBM to rotate laterally along the shaft; thus, the operation must be stopped immediately.

There are three kinds of angle analysis methods, including the Euler angle method, direction cosine method, and quaternion method [40]. By using the Euler angle method, if the reference coordinate rotates *α* around the *X* axis, then rotates *β* around the *Y* axis, and finally rotates *γ* around the *Z* axis, it can overlap with angle coordinate, and the transformation matrix $C_{r}^{a}$ after three rotations is expressed as follows:

(1) $$\begin{array}{l} C_{r}^{a}=C_{r}^{\alpha }C_{r}^{\beta }C_{r}^{\gamma }=\left[\begin{array}{ccc} 1 & 0 & 0\\ 0 & cos\alpha & sin\alpha \\ 0 & -sin\alpha & cos\alpha \end{array}\right]\left[\begin{array}{ccc} cos\beta & 0 & -sin\beta \\ 0 & 1 & 0\\ sin\beta & 0 & cos\beta \end{array}\right]\left[\begin{array}{ccc} cos\gamma & sin\gamma & 0\\ -sin\gamma & cos\gamma & 0\\ 0 & 0 & 1 \end{array}\right]\\ =\left[\begin{array}{ccc} cos\beta cos\gamma & cos\beta sin\gamma & -sin\beta \\ sin\alpha sin\beta cos\gamma -cos\alpha sin\gamma & sin\alpha sin\beta sin\gamma +cos\alpha cos\gamma & sin\alpha cos\beta \\ cos\alpha sin\beta cos\gamma +sin\alpha sin\gamma & cos\alpha sin\beta sin\gamma -sin\alpha cos\gamma & cos\alpha cos\beta \end{array}\right] \end{array}.$$

where *r* stands for the reference coordinate system, and *a* is on behalf of the angle coordinate system of the SBM. $C_{r}^{\alpha }$, $C_{r}^{\beta }$, $C_{r}^{\gamma }$, each, respectively, represents the transformation matrix of the reference coordinate rotation around the *X* axis, the *Y* axis, and the *Z* axis. $C_{r}^{a}$ is the orthogonal matrix, which is associated with the rotating order. Therefore, $C_{a}^{r}$ can be expressed as:

(2) $$C_{a}^{r}={\left(C_{r}^{a}\right)}^{-1}={\left(C_{r}^{a}\right)}^{T}.$$

The rotation transformation matrix represents the transformation relation between different coordinate systems, and can also be called the direction cosine matrix, $C_{a}^{r}$ is defined as:

(3) $$C_{a}^{r}=\left[\begin{array}{lll} C_{11} & C_{12} & C_{13}\\ C_{21} & C_{22} & C_{23}\\ C_{31} & C_{32} & C_{33} \end{array}\right].$$

Through the information of the rotation transformation matrix, the Euler angle can be solved as:

(4) $$\{\begin{array}{l} \alpha_{main}=arctan\left(\frac{C_{32}}{C_{33}}\right)\\ \beta_{main}=arcsin\left(-C_{31}\right)\\ \gamma_{main}=arctan\left(\frac{C_{21}}{C_{11}}\right) \end{array}.$$

where, $\alpha_{main}$, $\gamma_{main}$ are calculated by an inverse trigonometric function, the values should be corrected by the positive and negative judgment of $C_{11}$, $C_{21}$, $C_{32}$, $C_{33}$, and the final angle can be given by:

(5) $$\begin{array}{l} \alpha =\{\begin{array}{l} \alpha_{main}C_{33}>0\\ \alpha_{main}+180^{{}^{\circ}}C_{33}<0,C_{32}>0\\ \alpha_{main}-180^{{}^{\circ}}C_{33}<0,C_{32}<0 \end{array}\\ \beta =\beta_{main}\\ \gamma =\{\begin{array}{l} \gamma_{main}C_{11}>0,C_{21}>0\\ \gamma_{main}+360^{{}^{\circ}}C_{11}>0,C_{21}<0\\ \gamma_{main}+180^{{}^{\circ}}C_{11}<0 \end{array} \end{array}.$$

If $\omega ={\left[\omega_{x}\;\omega_{y}\;\omega_{z}\right]}^{T}$ is the angle velocity vector of the gyroscope in the angle coordinate system, and ${\left[\dot{\alpha }\dot{\;\beta \;}\dot{\gamma \;}\right]}^{T}$ represents the differential of the attitude angle, the relationship between them can be shown as:

(6) $$\left[\begin{array}{c} \dot{\alpha }\\ \dot{\beta }\\ \dot{\gamma } \end{array}\right]=\frac{1}{cos\beta }\left[\begin{array}{ccc} cos\beta & sin\alpha sin\beta & cos\alpha sin\beta \\ 0 & cos\alpha cos\beta & -sin\alpha cos\beta \\ 0 & sin\alpha & cos\alpha \end{array}\right]\left[\begin{array}{c} \omega_{x}\\ \omega_{y}\\ \omega_{z} \end{array}\right].$$

Because the analysis processes of the Euler angle method include the trigonometric function operation, the calculation speed is slow and the singularity problem will appear; thus, the Euler angle method cannot be used for the whole attitude measurement. Meanwhile, the direction cosine method solves the singularity problem, but the computation of the rotation matrix elements is still very large [39]. To solve these problems, the quaternion method can be applied in the attitude description of the SBM. The symbol *Q* usually identifies the rotation quaternion of the angle coordinate relative to the reference coordinate, *Q* can be expressed as a plural number consisting of a real number $q_{0}$ and three imaginary numbers: $q_{1}i$, $q_{2}$*j*, $q_{3}k$:

(7) $$Q=q_{0}+q_{1}i+q_{2}j+q_{3}k.$$

The quaternion form of the transformation matrix $C_{a}^{r}$ is:

(8) $$C_{a}^{r}=\left[\begin{array}{ccc} q_{0}^{2}+q_{1}^{2}-q_{2}^{2}-q_{3}^{2} & 2\left(q_{1}q_{2}-q_{0}q_{3}\right) & 2\left(q_{1}q_{3}+q_{0}q_{2}\right)\\ 2\left(q_{1}q_{2}+q_{0}q_{3}\right) & q_{0}^{2}-q_{1}^{2}+q_{2}^{2}-q_{3}^{2} & 2\left(q_{2}q_{3}-q_{0}q_{1}\right)\\ 2\left(q_{1}q_{3}-q_{0}q_{2}\right) & 2\left(q_{2}q_{3}+q_{0}q_{1}\right) & q_{0}^{2}-q_{1}^{2}-q_{2}^{2}+q_{3}^{2} \end{array}\right].$$

According to Equation (4), the attitude angle can be expressed as the quaternion form:

(9) $$\left\{ \begin{array}{l} \alpha_{main}=arctan\left[\frac{2\left(q_{2}q_{3}+q_{0}q_{1}\right)}{q_{0}^{2}-q_{1}^{2}-q_{2}^{2}+q_{3}^{2}}\right]\\ \beta_{main}=arcsin\left[-2\left(q_{1}q_{3}-q_{0}q_{2}\right)\right]\\ \gamma_{main}=arctan\left[\frac{2\left(q_{1}q_{2}+q_{0}q_{3}\right)}{q_{0}^{2}+q_{1}^{2}-q_{2}^{2}-q_{3}^{2}}\right] \end{array}\right..$$

The angle correction method adopted in Equation (5) is also applicable to Equation (9). The attitude angle of the initial state is substituted into Equation (10), and then the initial quaternions can be obtained.

(10) $$\{\begin{array}{l} q_{0}=cos(\beta /2)cos(\alpha /2)cos(\gamma /2)+sin(\beta /2)sin(\alpha /2)sin(\gamma /2)\\ q_{1}=sin(\beta /2)cos(\alpha /2)cos(\gamma /2)-cos(\beta /2)sin(\alpha /2)sin(\gamma /2)\\ q_{2}=cos(\beta /2)sin(\alpha /2)cos(\gamma /2)+sin(\beta /2)cos(\alpha /2)sin(\gamma /2)\\ q_{3}=cos(\beta /2)cos(\alpha /2)sin(\gamma /2)-sin(\beta /2)sin(\alpha /2)cos(\gamma /2) \end{array}.$$

In the working process of the SBM, its attitude changes over time, so the quaternions are also the variables about time [31]. According to the angle velocity vector $\omega ={\left[\omega_{x}\;\omega_{y}\;\omega_{z}\right]}^{T}$ and Equation (7), the quaternion differential equation is given as:

(11) $$\frac{dQ}{dt}=\frac{1}{2}Q\times \omega =\frac{1}{2}\left[\begin{array}{cccc} 0 & -\omega_{x} & -\omega_{y} & -\omega_{z}\\ \omega_{x} & 0 & \omega_{z} & -\omega_{y}\\ \omega_{y} & -\omega_{z} & 0 & \omega_{x}\\ \omega_{z} & \omega_{y} & -\omega_{x} & 0 \end{array}\right]\left[\begin{array}{l} q_{0}\\ q_{1}\\ q_{2}\\ q_{3} \end{array}\right]=\frac{1}{2}\Omega_{s}Q.$$

To ensure real-time data acquisition and reduce the measurement errors, the fourth order Runge-Kutta method is used to improve the sampling precision [41]. *T* is the sampling period, $Q_{t+2T}$, $\omega_{t+2T}$ are, respectively, the quaternion and angle velocity at time *t* + 2*T*, and $Q_{t}$, $\omega_{t}$ are the values at time *t*. The differential equation can be derived as:

(12) $$\{\begin{array}{l} Q_{k+2T}=Q_{t}+\frac{T}{3}\left(K_{1}+2K_{2}+2K_{3}+K_{4}\right)\\ K_{1}=\frac{1}{2}Q_{t}\omega_{t}\\ K_{2}=\frac{1}{2}\left(Q_{t}+K_{1}T\right)\omega_{t+T}\\ K_{3}=\frac{1}{2}\left(Q_{t}+K_{2}T\right)\omega_{t+T}\\ K_{4}=\frac{1}{2}\left(Q_{t}+2K_{3}T\right)\omega_{t+2T} \end{array}.$$

To calculate the attitude angle, the updated quaternions can be obtained through the angle velocity of the gyroscope in each calculation period. However, angle velocity errors accumulate over time, which leads to poor reliability in long-term measurement; thus, it is necessary to adopt other methods to compensate angle velocity errors.

### 3.2. Displacement Coordinate Analysis

**Figure 7:**
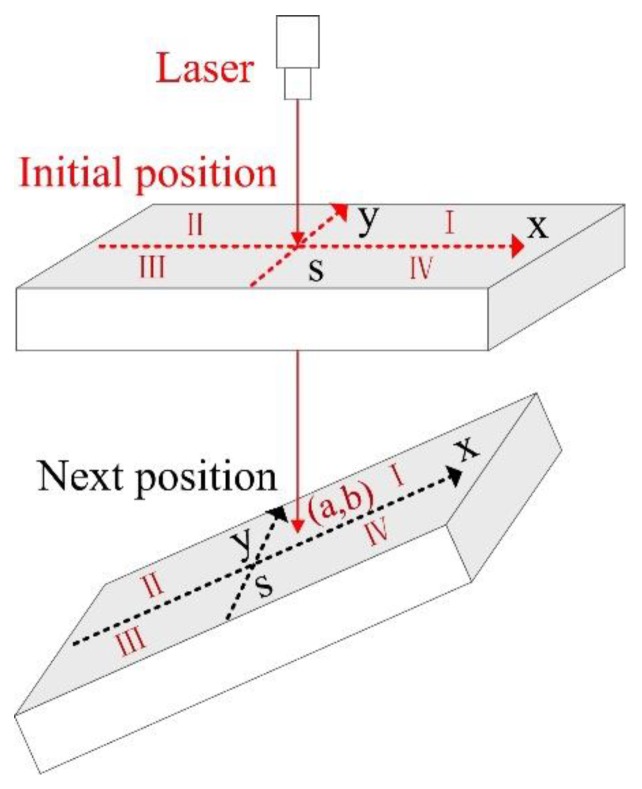
Displacement coordinate system of the SBM.

The displacement coordinate system is an effective supplement to the angle coordinate method, and it can accurately indicate the relative position of the SBM in a shaft. Before the drilling operation, the laser orientation instrument is installed vertically downward on the sidewall of the shaft. After the reference point *S* of the initial coordinate is confirmed in the power-on test, the PSD is fixed reliably at the designated position on the top of the SBM. As shown in Figure 7, when the displacement deviation appears in the downward drilling, the PSD can determine the light spot position (*a*, *b*) in the two-dimensional coordinate system. The displacement relative to point *S* can be defined as $d=\sqrt{a^{2}+b^{2}}$, and the threshold range of *d* should be determined according to the specific requirements of the construction. In particular, the values of *a* and *b* can clearly indicate the specific position of the point, and they are the important indexes of displacement coordinate analysis.

**Figure 8:**
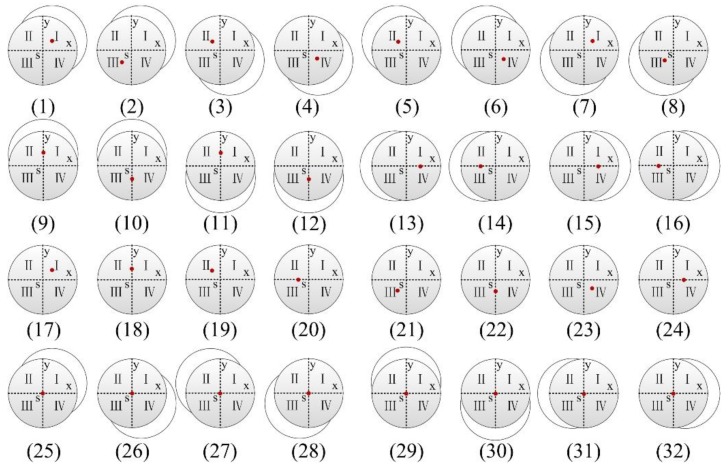
Top view of the SBM deviation.

As shown in Figure 8, each image stands for the one form of displacement deviation in actual engineering, and the red point represents the light spot position on the PSD. Case (1–8), respectively, mean the bottom deviation as the light spot is in different quadrants; case (9–16), respectively, show the displacement deviation when the light spot is on the coordinate axis; case (17–24), respectively, indicate that only the displacement changes without angle deflection; case (25–32), respectively, indicate that only the angle changes without relative displacement. In addition to the above cases, the deviation judgment should be combined with the yaw angle *γ*. When *γ* = 0, the deviation correction control system is likely to disorder and needs to troubleshoot in time; as *γ* ≠ 0, the system equipment or environment is abnormal, and the construction must stop.

Generally, the change of relative displacement is accompanied by the changes of the roll angle *α* and the pitch angle *β*. The relevant data should be statistically analyzed to accurately describe the deviation of the SBM, which will provide the decision basis for the effective implementation of the drilling operation plan and deviation correction control. The corresponding relationship between the attitude changes and coordinate values is shown in Table 1.

In fact, there are 81 combination forms through the coordinate values of *α*, *β*, *a*, *b*, but only 33 forms in Table 1 are normal states, and the others are abnormal conditions. To facilitate the subsequent analysis, the displacement error and speed error can be expressed as:

(13) $$\left\{ \begin{array}{l} e_{d}=d_{r}-d_{est}\\ e_{v}=V_{r}-V_{est} \end{array}\right..$$

where $e_{s}$, $e_{v}$ are, respectively, the displacement error and speed error, $d_{r}$, $V_{r}$ are, respectively, the real values of displacement and speed, and $d_{est}$, $V_{est}$, respectively, stand for the estimation values of displacement and speed. The detailed calculation processes of coordinate values and errors are introduced in Section 4.

## 4. Attitude Estimation Algorithms

In the attitude measurement system of the SBM, the relative displacement, rotation angle velocity, acceleration, and magnetic strength can be obtained through the PSD, barometer, gyroscope, accelerometer, and magnetometer. However, the attitude angle of the SBM cannot be directly acquired by these sensors. The attitude angle, displacement, and speed must be solved by the estimation algorithm, and then they can be used for attitude control. MEMS sensors are the key data acquisition units, and they inevitably have noises because of the manufacturing process, temperature, vibration, and other factors [42]. The error models should be established in the attitude calculation and corrected in the process of multi-sensor data fusion. The complementary filter is an analysis method applied in the frequency domain, whereas the Kalman filter is used to deal with the signals in the time domain. Under normal circumstances, useful signals and interfering noises overlap in the frequency and time domains, which leads to useful signals with a degree of randomness. Regarding the switching algorithm between the complementary filter and Kalman filter, the robust adaptive control [43] and multi-model robust control [44] are of some reference significance. However, the proposed algorithm is the combination of the two methods, and it is feasible in theory by combining the complementary filter with the EKF. Moreover, according to the characteristics of signals and noises, the error compensation models can be established to recover useful information and effectively improve the measurement accuracy and dynamic performance.

### 4.1. Complementary Filter

Due to the good dynamic response characteristics of gyroscopes, the attitude angle obtained by the integration is relatively accurate in a short period. However, the drift errors accumulate continuously over time and the accuracy decreases, so gyroscopes are not suitable for long-term measurement. At the same time, there are no time accumulation errors for accelerometers and magnetometers; they have good long-term stability, but they are not suitable for short-term measurement because of slow dynamic response, low short-time measurement accuracy, and are easily affected by the motion acceleration and the external environment. Obviously, the characteristics of the gyroscope, accelerometer, and magnetometer are complementary in the frequency domain, and the complementary filter can distinguish noise from the frequency domain, so the complementary characteristics can be used to obtain an accurate attitude angle by information fusion [35]. The transfer function of the complementary filter can be written as:

(14) $$\left\{ \begin{array}{l} \hat{C}(s)=C_{0}(s)G_{L}(s)+C_{\omega }(s)G_{H}(s)\approx C(s)\\ G_{L}(s)=C(s)/(s+C(s))\\ G_{H}(s)=s/(s+C(s))\\ C_{o}(s)=C(s)+\mu_{H}\\ C_{\omega }(s)=C(s)+\mu_{L} \end{array}\right..$$

where C(s) is the real attitude matrix, $\hat{C}\left(s\right)$ represents the attitude matrix of the complementary filter estimation, and the low-pass filter $G_{L}\left(s\right)$ is designed to remove the high-frequency noise $\mu_{H}$ of the accelerometer and magnetometer. In addition, the observation data matrix is $C_{o}\left(s\right)$, and the high-pass filter $G_{H}\left(s\right)$ is designed to remove the low-frequency noise $\mu_{L}$ of the gyroscope; the observation data matrix is $G_{\omega }\left(s\right)$ at this time. $G_{L}\left(s\right)+G_{H}\left(s\right)=1$ shows that there is no attenuation of the attitude signal.

To eliminate the influence of static errors, PI feedback control can be added on the basis of the complementary filter; thus, C(s) will be expressed as $C\left(s\right)=K_{p}+K_{i}/s$ [45], and $K_{p}$ determines the cut-off frequency $f_{T}$ of the filters ($f_{T}=K_{p}/2\pi )$. When the noise frequency $f>f_{T}$, the gyroscope plays a major role in the calculation results; as $f<f_{T}$, the results come from accelerometers and magnetometers. $K_{p}$ mainly affects the dynamic performance and stability of the complementary filter; a large $K_{p}$ value makes the adjustment time too long, which reduces the real-time performance of the algorithm, and the small $K_{p}$ value increases output error. Moreover, $K_{i}$ determines the time of the filter eliminating static errors, generally $K_{i}=\left(0.01-0.1\right)\;K_{p}$, $K_{p}=3$, $K_{i}=0.06$ in this paper.

The complementary filter is adopted to correct the roll angle and pitch angle data of the gyroscope by the accelerometer as well as compensate the yaw angle data by the magnetometer. The output values of the accelerometer and magnetometer in the angle coordinate system are respectively: $a_{s}={\left[a_{x}\;a_{y}\;a_{z}\right]}^{T}$, $m_{s}={\left[m_{x}\;m_{y}\;m_{z}\right]}^{T}$. When the accelerometer is stationary or moving at a constant speed relative to the reference coordinate system, the value of gravity acceleration is $a_{r}={\left[0\;0\;g\right]}^{T}$, the unit vector is ${\left[0\;0\;1\right]}^{T}$ [38]. The roll angle $\alpha_{a}$ and pitch angle $\beta_{a}$ solved by the accelerometer and the yaw angle $\gamma_{m}$ obtained from magnetometer can be derived as:

(15) $$\{\begin{array}{l} \alpha_{a}=arctan\left(a_{y}/a_{z}\right)\\ \begin{array}{l} \beta_{a}=arcsin\left(-a_{x}/g\right)\\ \gamma_{m}=arctan\left(\frac{m_{y}cos\alpha_{a}-m_{z}sin\alpha_{a}}{m_{x}cos\beta_{a}+m_{y}sin\alpha_{a}sin\beta_{a}+m_{z}cos\alpha_{a}sin\beta_{a}}\right) \end{array} \end{array}.$$

The value of the magnetometer is $H={\left[H_{n}\;0\;H_{d}\right]}^{T}$ in the reference coordinate system, $H_{n}$ is the north component of the geomagnetic field in the reference coordinate system, and $H_{d}$ is the vertical component; thus, the unit vector is ${\left[h_{n}\;0\;h_{d}\right]}^{T}$. The gravitational field measured by the accelerometer is converted to the angle coordinate system.

(16) $$a_{f}=\left[\begin{array}{l} a_{fx}\\ a_{fy}\\ a_{fz} \end{array}\right]=C_{r}^{a}\left[\begin{array}{c} 0\\ 0\\ 1 \end{array}\right]=\left[\begin{array}{c} 2\left(q_{1}q_{3}-q_{0}q_{2}\right)\\ -2\left(q_{2}q_{3}+q_{0}q_{1}\right)\\ q_{0}^{2}-q_{1}^{2}-q_{2}^{2}+q_{3}^{2} \end{array}\right].$$

The geomagnetic field measured by the magnetometer is converted to the angle coordinate system.

(17) $$m_{f}=\left[\begin{array}{c} m_{fx}\\ m_{fy}\\ m_{fz} \end{array}\right]=C_{r}^{a}\left[\begin{array}{c} h_{n}\\ 0\\ h_{d} \end{array}\right]=\left[\begin{array}{c} h_{n}\left(q_{0}^{2}+q_{1}^{2}-q_{2}^{2}-q_{3}^{2}\right)+2h_{d}\left(q_{1}q_{3}-q_{0}q_{2}\right)\\ 2h_{n}\left(q_{1}q_{2}-q_{0}q_{3}\right)+2h_{d}\left(q_{2}q_{3}+q_{0}q_{1}\right)\\ 2h_{n}\left(q_{1}q_{3}+q_{0}q_{2}\right)+h_{d}\left(q_{0}^{2}-q_{1}^{2}-q_{2}^{2}+q_{3}^{2}\right) \end{array}\right].$$

However, the error divergence occurs when the data status is updated, which will reduce the reliability of angle estimation, and the attitude transformation matrix of quaternions no longer satisfies the normalization after a long time, so the attitude transformation matrix should be normalized when the quaternions are updated every time. $a_{s}^{\prime }$, $m_{s}^{\prime }$, *Q*′ are, respectively, are the normalization form of the accelerometer measurement value $a_{s}$, the magnetometer measurement value $m_{s}$ and the quaternion *Q*; they are given as:

(18) $$\left\{ \begin{array}{l} a_{s}^{\prime }=\frac{1}{\sqrt{a_{x}^{2}+a_{y}^{2}+a_{z}^{2}}}{\left[\begin{array}{ccc} a_{x} & a_{y} & a_{z} \end{array}\right]}^{T}\\ m_{s}^{\prime }=\frac{1}{\sqrt{m_{x}^{2}+m_{y}^{2}+m_{z}^{2}}}{\left[\begin{array}{ccc} m_{x} & m_{y} & m_{z} \end{array}\right]}^{T}\\ Q^{\prime }=\frac{1}{\sqrt{q_{0}^{2}+q_{1}^{2}+q_{2}^{2}+q_{3}^{2}}}\left(q_{0}+q_{1}i+q_{2}j+q_{3}k\right) \end{array}\right..$$

where $a_{s}^{\prime }$, $m_{s}^{\prime }$ can, respectively, carry out the cross-product operation with $a_{f}$, $m_{f}$, and then $e_{\alpha \beta }$, $e_{\gamma }$ can be obtained, $e_{\alpha \beta }$ is the measurement error of roll angle *α*, and pitch angle *β*, $e_{\gamma }$ is the measurement error of yaw angle *γ*. Furthermore, the overall measurement error *e* can be expressed as:

(19) $$e=e_{\alpha \beta }+e_{\gamma }=a_{s}^{\prime }\times a_{f}+m_{s}^{\prime }\times m_{f}.$$

The compensation value *η* of the gyroscope drift can be obtained by the overall measurement error *e* by using PI feedback control.

(20) $$\eta =K_{P}e+K_{i}\int e.$$

Finally, $\omega =\omega_{s}+\eta$ is used to compensate the angle velocity $\omega_{s}$ of the gyroscope, and ω is substituted into Equation (12) to update quaternions iteratively; thus, the attitude angle ${\left[\alpha_{i}\;\beta_{i}\;\gamma_{i}\right]}^{T}$ is calculated preliminarily.

### 4.2. Extended Kalman Filter

The Kalman filter is an optimal linear estimation, and its principle is that the optimal value of the current state is estimated based on the statistical error at the previous moment and the error of the measured value at this moment; the attitude angle and displacement at the next moment are obtained through the prediction and update process. However, the filter model of the SBM is nonlinear, and the filtering algorithm must be suitable for a nonlinear system. The EKF is established on the basis of the Kalman filter, the specific process of the EKF is that the nonlinear part of the state equation is expanded into the Taylor series, after omitting the parts of the second order and above, the approximate linearized model is obtained and applied in the state estimation with a linear Kalman filter method.

To design the Kalman filter, the state equation and observation equation of the system need to be established. $X_{k}={\left[A_{k}\;d_{k}\;V_{k}\right]}^{T}$ is the state model variable, $A_{k}={\left[\alpha_{k}\;\beta_{k}\;\gamma_{k}\right]}^{T}$ is the attitude angle vector, and $A_{k}$ is calculated by the complementary filter method. $d_{k}={\left[d_{xk}\;d_{yk}\;d_{zk}\right]}^{T}$ is the displacement vector, $d_{xk}$, $d_{yk}$ are the horizontal displacements from PSD, and $d_{zk}$ is the vertical displacement solved by the barometer. Moreover, $V_{k}={\left[V_{xk}\;V_{yk}\;V_{zk}\right]}^{T}$ is the speed vector. According to the kinematics theory, the relations of different vectors are expressed as:

(21) $$\begin{array}{l} \left[\begin{array}{c} {\dot{V}}_{xk}\\ {\dot{V}}_{yk}\\ {\dot{V}}_{zk} \end{array}\right]=\left[\begin{array}{l} a_{xk}\\ a_{yk}\\ a_{zk} \end{array}\right]=C_{a}^{r}\left[\begin{array}{l} a_{x}-w_{ax}\\ a_{y}-w_{ay}\\ a_{z}-w_{az} \end{array}\right]-\left[\begin{array}{l} 0\\ 0\\ g \end{array}\right]\\ \left[\begin{array}{c} {\dot{d}}_{xk}\\ {\dot{d}}_{yk}\\ {\dot{d}}_{zk} \end{array}\right]=\left[\begin{array}{c} V_{xk}\\ V_{yk}\\ V_{zk} \end{array}\right] \end{array}.$$

where ${\left[a_{xk}\;a_{yk}\;a_{zk}\right]}^{T}$ is the motion acceleration vector, and $C_{a}^{r}$ is the transfer matrix from the angle coordinate system to the reference coordinate system. ${\left[w_{ax}\;w_{ay}\;w_{az}\right]}^{T}$ is the noise vector of the accelerometer in the axial direction, and its value is available from the accelerometer manual. The measurement value ${\left[a_{x}\;a_{y}\;a_{z}\right]}^{T}$ of the accelerometer is the vector sum of gravitational acceleration and motion acceleration, and it is necessary to compensate the gravitational acceleration when the motion acceleration is calculated. In practical application, the attitude angle, displacement, and vertical speed are the key indexes; other parameters are only process calculation variables. The interval time of system status update is *T*, on the basis of the differential equation of the attitude angle and kinematics analysis, the state equation of Kalman filter is obtained as:

(22) $$\begin{array}{l} X_{k}=f\left(k-1,X_{k-1}\right)+w_{k-1}=\\ \left[\begin{array}{c} \alpha_{k-1}\\ \beta_{k-1}\\ \gamma_{k-1}\\ d_{x(k-1)}\\ d_{y(k-1)}\\ d_{z(k-1)}\\ V_{z(k-1)} \end{array}\right]+\left[\begin{array}{ccccccc} 1 & sin\alpha_{k-1}\tan\beta_{k-1} & cos\alpha_{k-1}\tan\beta_{k-1} & 0 & 0 & 0 & 0\\ 0 & cos\alpha_{k-1} & -sin\alpha_{k-1} & 0 & 0 & 0 & 0\\ 0 & sin\alpha_{k-1}\sec\beta_{k-1} & cos\alpha_{k-1}\sec\beta_{k-1} & 0 & 0 & 0 & 0\\ 0 & 0 & 0 & 1 & 0 & 0 & 0\\ 0 & 0 & 0 & 0 & 1 & 0 & 0\\ 0 & 0 & 0 & 0 & 0 & 1 & 0\\ 0 & 0 & 0 & 0 & 0 & 0 & 1 \end{array}\right]\left[\begin{array}{c} \omega_{x(k-1)}\\ \omega_{y(k-1)}\\ \omega_{z(k-1)}\\ V_{x(k-1)}\\ V_{y(k-1)}\\ V_{z(k-1)}\\ a_{z(k-1)} \end{array}\right]T+\left[\begin{array}{c} w_{\alpha (k-1)}\\ w_{\beta (k-1)}\\ w_{\gamma (k-1)}\\ w_{dx(k-1)}\\ w_{dy(k-1)}\\ w_{dz(k-1)}\\ w_{az(k-1)} \end{array}\right] \end{array}.$$

where ${\left[w_{\alpha \left(k-1\right)}\;w_{\beta \left(k-1\right)}\;w_{\gamma \left(k-1\right)}w_{dx\left(k-1\right)}\;w_{dy\left(k-1\right)}\;w_{dz\left(k-1\right)}\;w_{az\left(k-1\right)}\right]}^{T}$ is the system noise vector. At the current moment, ${\left[\alpha_{ak}\;\beta_{ak}\;\gamma_{mk}\right]}^{T}$ is the attitude angle calculated by accelerometer and magnetometer, and the speed and displacement are solved by the integral of motion acceleration. Based on these observational variables, the observation equation is expressed as:

(23) $$Z_{k}=h\left(k,X_{k}\right)+\sigma_{k}=H_{k}\left[\begin{array}{c} \alpha_{ak}\\ \beta_{ak}\\ \gamma_{mk}\\ d_{xk}\\ d_{yk}\\ d_{zk}\\ V_{zk} \end{array}\right]+\sigma_{k}=\left[\begin{array}{ccccccc} 1 & 0 & 0 & 0 & 0 & 0 & 0\\ 0 & 1 & 0 & 0 & 0 & 0 & 0\\ 0 & 0 & 1 & 0 & 0 & 0 & 0\\ 0 & 0 & 0 & 1 & 0 & 0 & 0\\ 0 & 0 & 0 & 0 & 1 & 0 & 0\\ 0 & 0 & 0 & 0 & 0 & 1 & 0\\ 0 & 0 & 0 & 0 & 0 & 0 & 1 \end{array}\right]\left[\begin{array}{c} \alpha_{ak}\\ \beta_{ak}\\ \gamma_{mk}\\ d_{xk}\\ d_{yk}\\ d_{zk}\\ V_{zk} \end{array}\right]+\left[\begin{array}{c} \sigma_{\alpha k}\\ \sigma_{\beta k}\\ \sigma_{\gamma k}\\ \sigma_{dxk}\\ \sigma_{dyk}\\ \sigma_{dzk}\\ \sigma_{Vzk} \end{array}\right].$$

where ${\left[\sigma_{\alpha k}\;\sigma_{\beta k}\;\sigma_{\gamma k}\;\sigma_{dxk}\;\sigma_{dyk}\;\sigma_{dzk}\;\sigma_{Vzk}\right]}^{T}$ is the observed noise vector. By using the EKF, the nonlinear part of the state equation is expanded by the Taylor series and approximated by the first order, and the approximate linearized model is obtained; thus, the Kalman filter can be applied. After the state equation linearization, the Jacobian matrix $\Phi_{k/k-1}$ can be derived as:

(24) $$\begin{array}{l} \Phi_{k/k-1}=\frac{\partial f\left(X_{k-1}\right)}{\partial X_{k-1}}=\\ \left[\begin{array}{ccccccc} 1+\frac{cos\alpha_{k-1}sin\beta_{k-1}}{cos\beta_{k-1}}\omega_{yk}T-\frac{sin\alpha_{k-1}sin\beta_{k-1}}{cos\beta_{k-1}}\omega_{zk}T & \frac{sin\alpha_{k-1}}{cos\beta_{k-1}^{2}}\omega_{yk}T+\frac{cos\alpha_{k-1}}{cos\beta_{k-1}^{2}}\omega_{zk}T & 0 & 0 & 0 & 0 & 0\\ -sin\alpha_{k-1}\omega_{yk}T-cos\alpha_{k-1}\omega_{zk}T & 1 & 0 & 0 & 0 & 0 & 0\\ \frac{cos\alpha_{k-1}}{cos\beta_{k-1}}\omega_{yk}T-\frac{sin\alpha_{k-1}}{cos\beta_{k-1}}\omega_{zk}T & \frac{sin\alpha_{k-1}sin\beta_{k-1}}{cos\beta_{k-1}^{2}}\omega_{yk}T+\frac{cos\alpha_{k-1}sin\beta_{k-1}}{cos\beta_{k-1}^{2}}\omega_{zk}T & 1 & 0 & 0 & 0 & 0\\ 0 & 0 & 0 & 1 & 0 & 0 & 0\\ 0 & 0 & 0 & 0 & 1 & 0 & 0\\ 0 & 0 & 0 & 0 & 0 & 1 & 0\\ 0 & 0 & 0 & 0 & 0 & 0 & 1 \end{array}\right] \end{array}.$$

The prediction and update process of the EKF algorithm is divided into five parts; they are as follows:

1. State prediction equation:
  (25) $${\hat{X}}_{k/k-1}=\Phi_{k/k-1}{\hat{X}}_{k-1}.$$
  ${\hat{X}}_{k/k-1}$ is the estimation of the status variable ${\hat{X}}_{k}$, $\Phi_{k/k-1}$ is the state transition matrix, i.e., the Jacobian matrix.
2. Covariance prediction equation:
  (26) $$P_{k/k-1}=\Phi_{k/k-1}P_{k-1}\Phi_{k/k-1}^{T}+Q_{k}.$$
  $P_{k/k-1}$ is the estimation of the state covariance matrix $P_{k-1}$, $Q_{k}$ is the covariance matrix of the system noise.
3. Kalman filter gain update:
  (27) $$K_{k}=P_{k/k-1}H_{k}^{T}/\left(H_{k}P_{k/k-1}H_{k}^{T}+R_{k}\right).$$
  $R_{k}$ is the covariance matrix of the observed noise.
4. Status update:
  (28) $${\hat{X}}_{k}={\hat{X}}_{k/k-1}+K_{k}\left(Z_{k}-H_{k}{\hat{X}}_{k/k-1}\right).$$
5. State covariance matrix update:
  (29) $$P_{k}=\left(I-K_{k}H_{k}\right)P_{k/k-1}.$$

**Figure 9:**
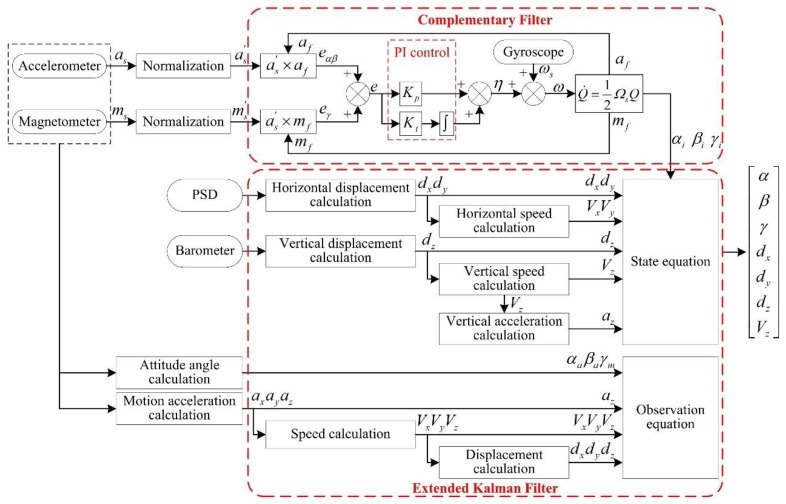
Flowchart of the proposed method combining the complementary filter with the extended Kalman filter (EKF).

In conclusion, based on the multi-sensor data of the gyroscope, accelerometer, magnetometer, PSD, and barometer, the fusion method of the complementary filter and EKF is used to calculate the displacement, attitude angle, and vertical movement speed, and the flowchart of the proposed method is shown in Figure 9.

## 5. Experiments and Discussion

### 5.1. Experimental Setup

**Figure 10:**
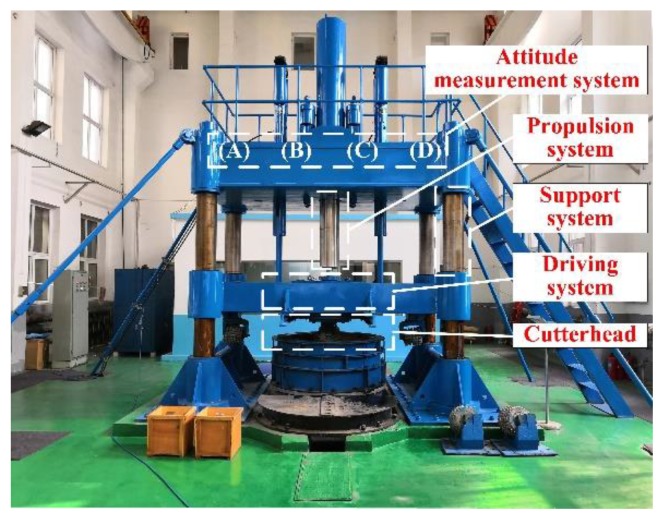
Experimental platform of attitude measurement system.

To verify the application effect of different methods, the experimental platform is designed for attitude measurement. As shown in Figure 10, the key parts of this platform are similar to the ones of the SBM, and the platform can simulate the normal working process of the SBM. The attitude measurement units are located at (A) and (D), and the embedded monitoring terminal and PSD are, respectively, in position (B) and (C). The comparative experiments are carried out for the complementary filter, EKF, and proposed filter method, and the experiment processes include the steady-state response test and dynamic response test. The sampling frequency is 50 Hz, and the test time is 40 s. The whole platform is kept level during the experiment, i.e., the reference values of the attitude angles are 0 degrees.

### 5.2. Steady-State Response Performance

**Figure 11:**
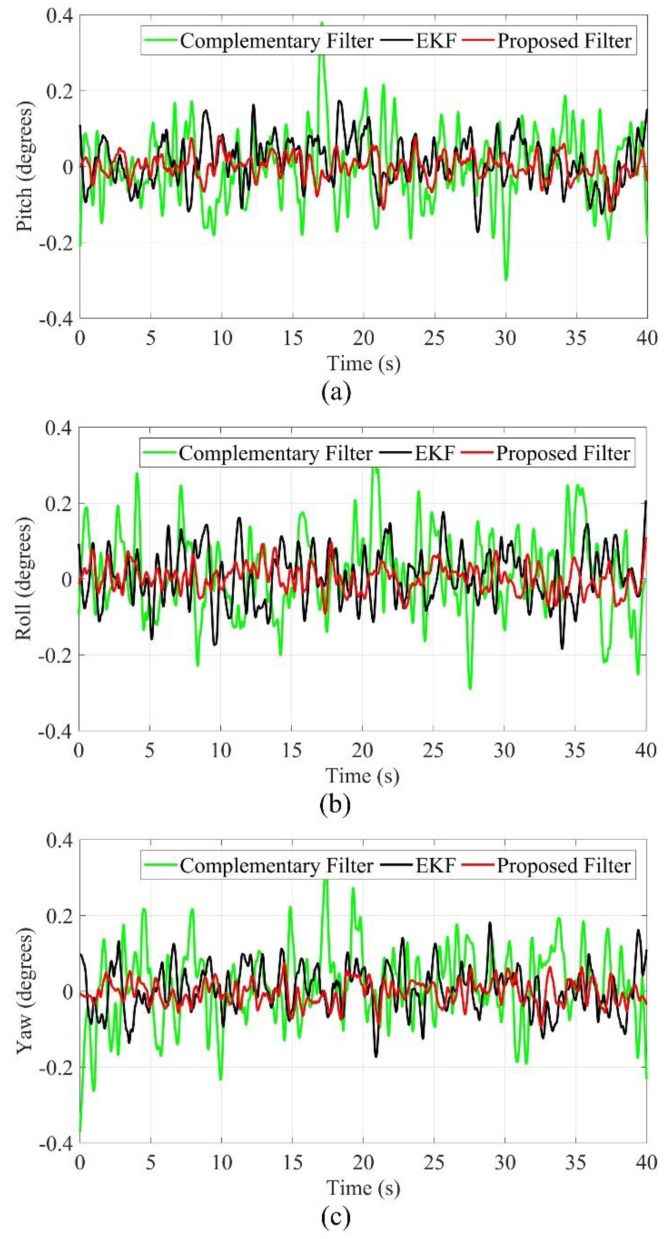
Static test (**a**) pitch angle; (**b**) roll angle; (**c**) yaw angle.

When the experimental platform is in a stationary state, the influences come from the external environment without the internal interference of the system. As shown in Figure 11, the measurement value of the PSD is 0 cm, the filtering function does not work, and the estimation errors of different methods are maintained in the range of −0.3–0.3 degrees, so all methods can meet the practical requirements.

**Figure 12:**
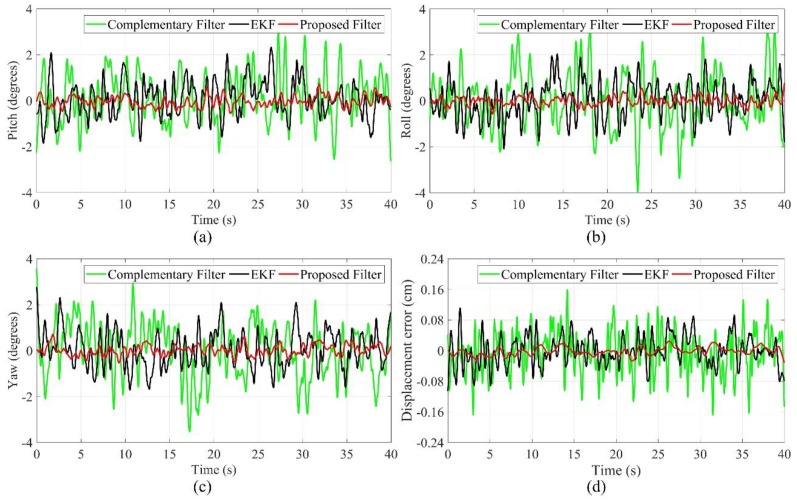
Drilling test at low speed (**a**) pitch angle; (**b**) roll angle; (**c**) yaw angle; (**d**) displacement error.

The speed of the cutterhead is 10 r/min during the drilling test at low speed, and the propulsion system makes the cutterhead remain in the rock breaking state. As shown in Figure 12, compared with the static test process, the mechanical vibration and electrical magnetic field have affected the attitude measurement, and the estimation errors increase obviously. The angle estimation errors of the EKF and complementary filter change in the range of −3–3 degrees, and the displacement estimation errors of the EKF and complementary filter change in the range of −0.16–0.16 cm; the estimation errors of the complementary filter are larger. However, the angle estimation errors of the proposed filter are in the range of −1–1 degrees, and the displacement estimation errors of the proposed filter change in the range of −0.04–0.04 cm, so the proposed filter has better performance.

**Figure 13:**
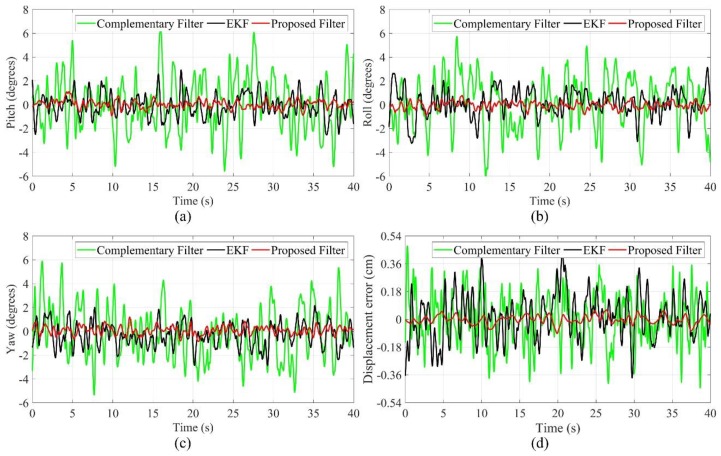
Drilling test at high speed (**a**) pitch angle; (**b**) roll angle; (**c**) yaw angle; (**d**) displacement error.

The speed of cutterhead is 40 r/min during the drilling test at high speed. As shown in Figure 13, compared with the low-speed drilling process, the estimation errors increase again. The angle estimation errors of the EKF and complementary filter are in the range of −6–6 degrees, and the displacement estimation errors of the EKF and complementary filter are in the range of −0.54–0.54 cm. Meanwhile, the angle estimation errors of the proposed filter are in the range of −2–2 degrees, and the displacement estimation errors of the proposed filter are in the range of −0.1–0.1 cm.

### 5.3. Dynamic Response Performance

**Figure 14:**
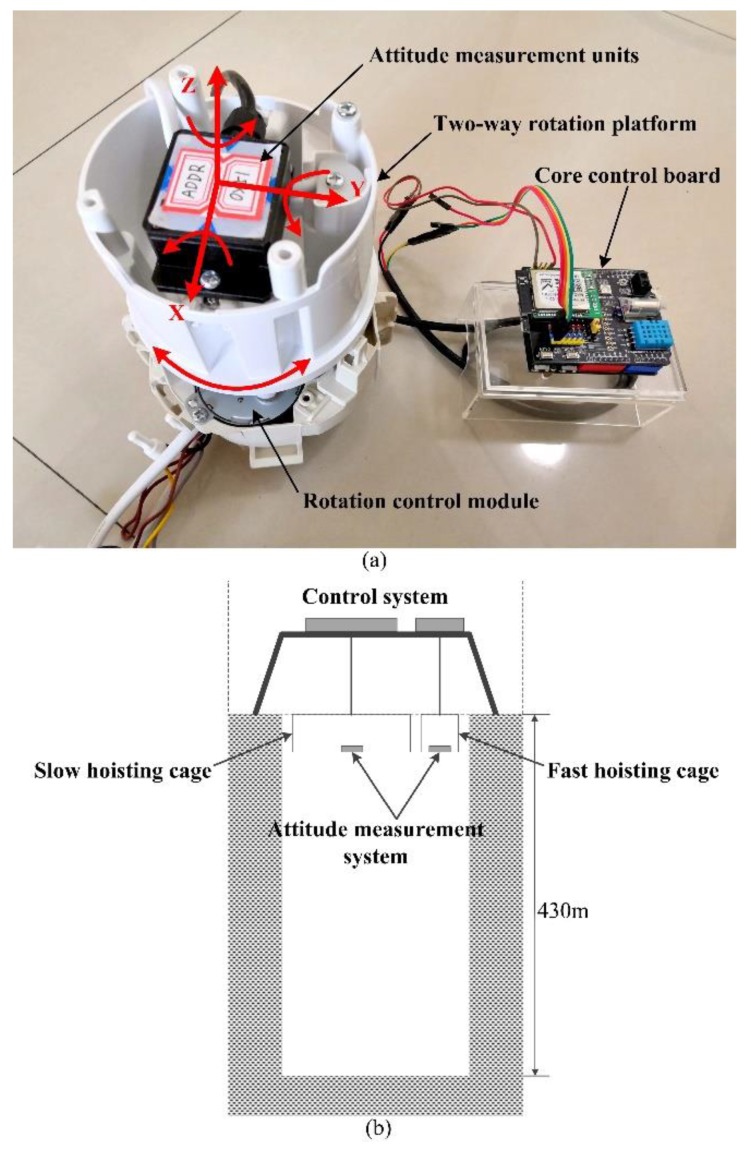
(**a**) Experiment equipment of dynamic response; (**b**) experimental environment of vertical tracking.

When the experimental platform maintains in the horizontal attitude, all of the test processes show the steady-state response performance of each algorithm in the normal drilling without deviation. However, the platform does not have the testing ability of deviation state and vertical movement. To verify the dynamic response performance of different algorithms, i.e., the performance of fast tracking the change of the attitude angle, we designed the test equipment for dynamic response. As shown in Figure 14a, it mainly includes attitude measurement units, a two-way rotation platform, core control board, and rotation control module. The sampling frequency of the test is 50 Hz, and the test time is 80 s. As shown in Figure 14b, the shaft hoisting system of a coal mine is selected as the experimental environment for tracking the vertical displacement and speed, and the system is composed of a slow hoisting cage, fast hoisting cage, and control system, and the shaft depth is 430 m.

**Figure 15:**
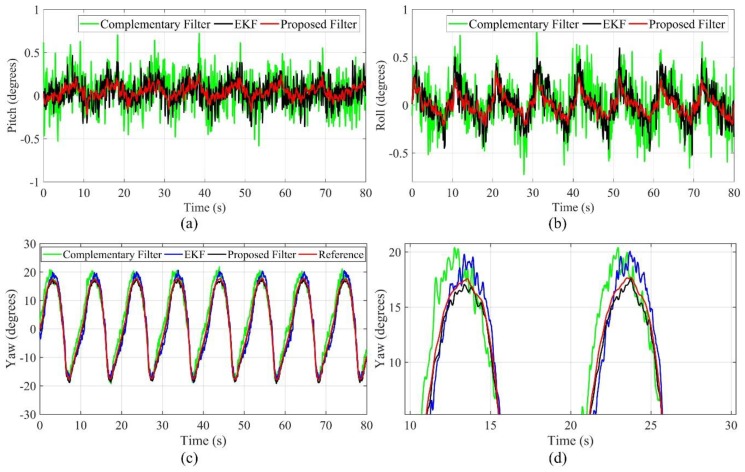
Estimated values of dynamic response test (**a**) pitch angle; (**b**) roll angle; (**c**) yaw angle; (**d**) part details of yaw angle.

In the drilling process, the deviation of the SBM is often accidental, so it is very important to measure the dynamic response performance of each algorithm, i.e., whether the algorithm can track the change of the attitude angle quickly and accurately. As shown in Figure 15, during the test, the roll angle and pitch angle remain at 0 degrees, and the yaw angle changes in the range of −17–17 degrees because of the rotation control module. Influenced by the mechanical vibration and electromagnetic interference of the motor, both of roll angle and pitch angle have a fluctuation at different degrees, but they are consistent with the change in the trend of the yaw angle. For the roll angle and pitch angle, the estimation errors of the EKF and complementary filter are in the range of −0.8–0.8 degrees, and the estimation errors of the proposed filter are in the range of −0.3–0.3 degrees. Meanwhile, for the yaw angle, the estimation errors of the EKF and complementary filter are in the range of −4–4 degrees, and the estimation errors of the proposed filter are in the range of −1.5–1.5 degrees. Therefore, the dynamic response performance of the proposed filter is better than the EKF and complementary filter.

**Figure 16:**
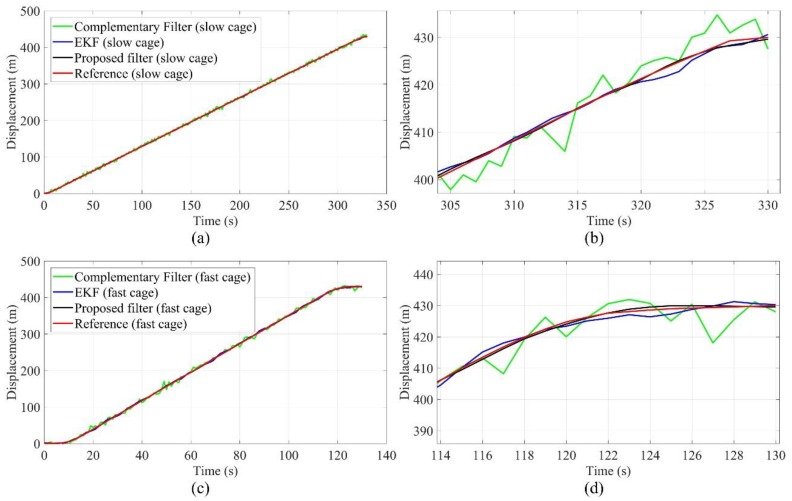
Displacement tracking test of the hoisting cage (**a**) displacement tracking of the slow cage; (**b**) part details of displacement tracking for the slow cage; (**c**) displacement tracking of the fast cage; (**d**) part details of displacement tracking for the fast cage.

**Figure 17:**
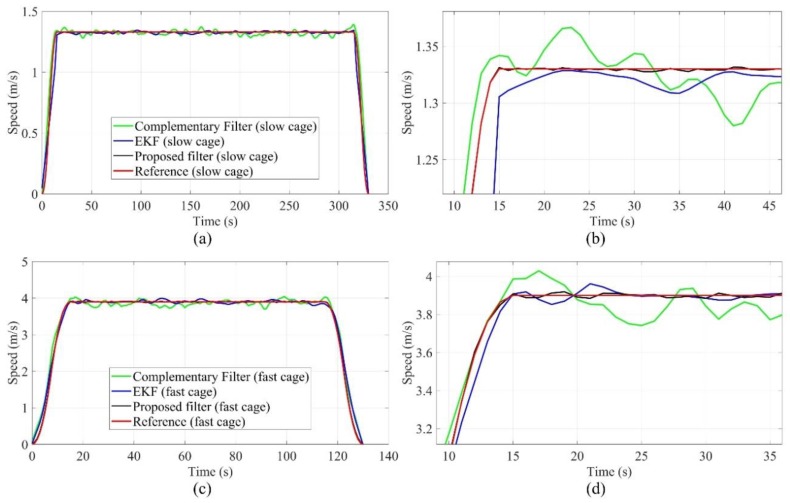
Speed tracking test of the hoisting cage (**a**) speed tracking of the slow cage; (**b**) part details of speed tracking for the slow cage; (**c**) speed tracking of the fast cage; (**d**) part details of speed tracking for the fast cage.

The displacement in the horizontal direction indicates the deviation degree of the SBM relative to the designed axis of the shaft, and the displacement in the vertical direction demonstrates the movement of support structure relative to the initial point. According to the control commands, the slow movement of the SBM is the normal process, and the abrupt changes of vertical displacement and speed are often caused by the instability of the support structure or geological structure, which are the important reference indexes for safe construction. Therefore, the accurate tracking and estimation for vertical displacement and speed is an indispensable function of the attitude measurement system. The vertical displacement (height) is converted from the barometer data, and the speed can be calculated, then the error is compensated by the proposed filter. The tracking performance of vertical displacement and speed can be simulated by the shaft hoisting system. As shown in Figure 16 and Figure 17, to ensure the safety of the hoisting cage, the whole operation process is divided into three stages, including the start and acceleration (time consumption: 15 s), running at constant speed (slow hoisting: 300 s, fast hoisting: 100 s), and slow stop (time consumption: 15 s). The maximum speed of the fast hoisting cage is 3.90 m/s, the maximum speed of the slow hoisting cage is 1.33 m/s, and the reference values of vertical displacement and speed are obtained by the hoisting control system. In the tracking process of the fast hoisting cage, the displacement tracking errors of the EKF and complementary filter are in the range of −10–10 m, and the speed tracking errors of the EKF and complementary filter are in the range of −0.3–0.3 m/s; the displacement tracking errors of the proposed filter are in the range of −2–2 m, and the speed tracking errors of the proposed filter are in the range of −0.15–0.15 m/s. Meanwhile, in the tracking process of the slow hoisting cage, the displacement tracking errors of the EKF and complementary filter are in the range of −8–8 m, and the speed tracking errors of the EKF and complementary filter are in the range of −0.2–0.2 m/s; the displacement tracking errors of the proposed filter are in the range of −1–1 m, and the speed tracking errors of the proposed filter are in the range of −0.1–0.1 m/s. During the process switching, the acceleration changes greatly, and the relative error is large. The errors of the constant speed process are mainly caused by the contact vibration between the guide wheel and guide rail structure; the faster the speed is, the more obvious the impact is. The comparison results show that the proposed filter has good tracking performance for displacement and speed, as well as the stability and robustness of the proposed filter is better than the simple complementary filter and traditional EKF.

To facilitate the statistical analysis, the mean variance of each method is calculated. As shown in Table 2, Table 3 and Table 4, in the steady-state response test, the maximum error of the attitude angle estimation is 3.24 degrees with the complementary filter, and the maximum error of the attitude angle estimation is 2.47 degrees for the EKF, then the maximum error of the attitude angle estimation is 0.75 degrees by the proposed filter; the maximum error of displacement estimation is 0.24 cm with the complementary filter, and the maximum error of displacement estimation is 0.15 cm for the EKF, then the maximum error of displacement estimation is 0.07 cm by the proposed filter. In the dynamic response test, the maximum error of the attitude angle estimation is 3.52 degrees with the complementary filter, and the maximum error of the attitude angle estimation is 2.73 degrees for the EKF, then the maximum error of the attitude angle estimation is 1.16 degrees by the proposed filter; the maximum error of displacement tracking is 4.64 m with the complementary filter, and the maximum error of displacement tracking is 3.22 m for the EKF, then the maximum error of displacement tracking is 1.36 m by the proposed filter; the maximum error of speed tracking is 0.23 m/s with the complementary filter, and the maximum error of speed tracking is 0.17 m/s for the EKF, then the maximum error of speed tracking is 0.06 m/s by the proposed filter. Obviously, due to the combination of the frequency domain and time domain method, the proposed filter gives full play to the advantages of tracking accuracy from the EKF and fast dynamic response from the complementary filter, and it can effectively suppress the disturbance errors. The steady-state response performance and dynamic response performance of the proposed filter are better than the EKF and complementary filter, so the proposed filter is suitable for the attitude measurement system of the SBM. Meanwhile, the complementary filter model is relatively simple in structure and small in computation. The calculation amount of the EKF and proposed filter varies a little, but they have higher requirements for hardware configuration. If there is a certain hardware condition, the proposed filter is a better choice on the premise of considering dynamic response, estimation accuracy, stability, and robustness.

To study the applicability and efficiency of related algorithms on different microcontrollers, the complementary filter, EKF, and the proposed filter are, respectively, implemented in TMS320F28335 (32-bit), STM32F051 (32-bit), MSP430F5418 (16-bit), and ATmega128 (8-bit). Without other subroutines running, the quantitative comparative analysis is carried out for the time consumption of different algorithms. As shown in Table 5, the 32-bit controllers can quickly complete the calculation process of three algorithms and meet the real-time requirements. However, MSP430F5418 and ATmega128 can only efficiently solve the complementary filter, which shows that the complementary filter requires little computation, and it is a good choice when the microcontroller configuration is low, but the dynamic error is large. Therefore, the 32-bit microcontroller is the basic configuration to meet the requirements of measurement error and dynamic response performance. Moreover, the updated efficiency of the MEMS sensors, data acquisition, and subroutine running will also take a certain time, so the microcontroller selection must leave enough margins.

## 6. Conclusions and Future Work

This paper presents a dual coordinate method suitable for the attitude measurement of the SBM, and the key parts of coordinate analysis are the attitude matrix transformation and state update, and they are in the form of quaternions to improve the efficiency of the calculation process. The dual coordinate method makes full use of the data from the gyroscope, accelerometer, magnetometer, PSD, and barometer to calculate the angle, displacement, and speed, which can accurately describe the attitude characteristics of the SBM in the restricted space of the shaft. Meanwhile, to improve the environmental adaptability of the attitude measurement system, we proposed the improved attitude estimation and error compensation method and deduced the method implementation process theoretically.

Through simulation experiments of the attitude estimation, this paper analyzes the steady-state performance and dynamic response performance of different algorithms. The mechanical vibration, electromagnetic interference, and random noise have significant effects on attitude estimation, but the air pressure effect is small. As a method of combining the time domain and frequency domain, the proposed filter can give full play to the advantages of the complementary filter and EKF, and it can ensure the effective acquisition of real attitude information. Furthermore, the time consumption experiments indicate that the complexity of different algorithms have a certain requirements for the hardware configuration, thus the experimental results can provide a reference for the follow-up research.

All experiments in this paper are performed at room temperature. Actually, the influence of temperature change on attitude estimation cannot be ignored, so it is necessary to study the temperature estimation and error compensation method. Moreover, due to the limitations of experimental conditions, there are still some differences between the simulation experiment platform and the SBM; thus, the method proposed in this paper still needs to be improved by the actual working test of the SBM.

## Figures and Tables

**Table 1:** Relationship between the attitude changes and coordinate values.

Light Spot Position	Bottom Deviation of SBM (Serial Numbers in Figure 7)	Angle Coordinate Values	Displacement Coordinate Values
Quadrant I	Upper right (1)	*β* > 0, *α* > 0	*a* > 0, *b* > 0
Quadrant I	Left lower (7)	*β* < 0, *α* < 0	*a* > 0, *b* > 0
Quadrant I	Only displacement deviation (17)	*β* = 0, *α* = 0	*a* > 0, *b* > 0
Quadrant II	Right lower (3)	*β* > 0, *α* < 0	*a* < 0, *b* > 0
Quadrant II	Upper left (5)	*β* < 0, *α* > 0	*a* < 0, *b* > 0
Quadrant II	Only displacement deviation (19)	*β* = 0, *α* = 0	*a* < 0, *b* > 0
Quadrant III	Upper right (2)	*β* > 0, *α* > 0	*a* < 0, *b* < 0
Quadrant III	Left lower (8)	*β* < 0, *α* < 0	*a* < 0, *b* < 0
Quadrant III	Only displacement deviation (21)	*β* = 0, *α* = 0	*a* < 0, *b* < 0
Quadrant IV	Right lower (4)	*β* > 0, *α* < 0	*a* > 0, *b* < 0
Quadrant IV	Upper left (6)	*β* < 0, *α* > 0	*a* > 0, *b* < 0
Quadrant IV	Only displacement deviation (23)	*β* = 0, *α* = 0	*a* > 0, *b* < 0
Positive half of X axis	Left (13)	*β* < 0, *α* = 0	*a* > 0, *b* = 0
Positive half of X axis	Right (15)	*β* > 0, *α* = 0	*a* > 0, *b* = 0
Positive half of X axis	Only displacement deviation (24)	*β* = 0, *α* = 0	*a* > 0, *b* = 0
Negative half of X axis	Left (14)	*β* < 0, *α* = 0	*a* < 0, *b* = 0
Negative half of X axis	Right (16)	*β* > 0, *α* = 0	*a* < 0, *b* = 0
Negative half of X axis	Only displacement deviation (20)	*β* = 0, *α* = 0	*a* < 0, *b* = 0
Positive half of Y axis	Up (9)	*β* = 0, *α* > 0	*a* = 0, *b* > 0
Positive half of Y axis	Down (11)	*β* = 0, *α* < 0	*a* = 0, *b* > 0
Positive half of Y axis	Only displacement deviation (18)	*β* = 0, *α* = 0	*a* = 0, *b* > 0
Negative half of Y axis	Up (10)	*β* = 0, *α* > 0	*a* = 0, *b* < 0
Negative half of Y axis	Down (12)	*β* = 0, *α* < 0	*a* = 0, *b* < 0
Negative half of Y axis	Only displacement deviation (22)	*β* = 0, *α* = 0	*a* = 0, *b* < 0
Central point S	Only angle deviation (upper right) (25)	*β* > 0, *α* > 0	*a* = 0, *b* = 0
Central point S	Only angle deviation (right lower) (26)	*β* > 0, *α* < 0	*a* = 0, *b* = 0
Central point S	Only angle deviation (upper left) (27)	*β* < 0, *α* > 0	*a* = 0, *b* = 0
Central point S	Only angle deviation (left lower) (28)	*β* < 0, *α* < 0	*a* = 0, *b* = 0
Central point S	Only angle deviation (up) (29)	*β* = 0, *α* > 0	*a* = 0, *b* = 0
Central point S	Only angle deviation (down) (30)	*β* = 0, *α* < 0	*a* = 0, *b* = 0
Central point S	Only angle deviation (left) (31)	*β* < 0, *α* = 0	*a* = 0, *b* = 0
Central point S	Only angle deviation (right) (32)	*β* > 0, *α* = 0	*a* = 0, *b* = 0
Central point S	No deviation	*β* = 0, *α* = 0	*a* = 0, *b* = 0

**Table 2:** RMS errors of different methods (attitude angles).

Attitude Angles (°)	Complementary Filter	EKF	Proposed Filter
Pitch (static)	0.07	0.05	0.02
Roll (static)	0.08	0.07	0.04
Yaw (static)	0.12	0.11	0.07
Pitch (low speed)	1.93	1.54	0.26
Roll (low speed)	1.87	1.48	0.24
Yaw (low speed)	2.11	1.75	0.37
Pitch (high speed)	3.13	2.42	0.51
Roll (high speed)	3.24	2.35	0.56
Yaw (high speed)	3.17	2.47	0.75
Pitch (dynamic response)	0.35	0.26	0.14
Roll (dynamic response)	0.47	0.38	0.23
Yaw (dynamic response)	3.52	2.73	1.16

**Table 3:** RMS errors of different methods (displacement).

Displacement Errors	Complementary Filter	EKF	Proposed Filter
Drilling at low speed	0.11 cm	0.06 cm	0.02 cm
Drilling at high speed	0.24 cm	0.15 cm	0.07 cm
Displacement tracking of fast cage	4.64 m	3.22 m	1.36 m
Displacement tracking of slow cage	3.37 m	2.15 m	0.53 m

**Table 4:** RMS errors of different methods (speed).

Speed Errors (m/s)	Complementary Filter	EKF	Proposed Filter
Speed tracking of fast cage	0.23	0.17	0.06
Speed tracking of slow cage	0.16	0.11	0.03

**Table 5:** Time consumption comparisons of different algorithms in microcontrollers.

Algorithms	TMS320F28335	STM32F051	MSP430F5418	ATmega128
Complementary Filter	0.08 ms	2.45 ms	47.37 ms	232.53 ms
EKF	2.35 ms	57.32 ms	1.15 s	5.21 s
Proposed Filter	2.46 ms	61.21 ms	1.26 s	5.87 s

## Nomenclature

The following abbreviations are used in this manuscript:

MEMS	Micro Electro Mechanical System
IMU	Inertial Measurement Unit
SBM	Shaft Boring Machine
EKF	Extended Kalman Filter
AHRS	Attitude and Heading Reference System
PSD	Position Sensitive Detector
RISC	Reduced Instruction Set Computing
ARM	Advanced RISC Machine
FPGA	Field Programmable Gate Array
DSP	Digital Signal Processor
